# *bifA* Regulates Biofilm Development of *Pseudomonas putida* MnB1 as a Primary Response to H_2_O_2_ and Mn^2+^

**DOI:** 10.3389/fmicb.2018.01490

**Published:** 2018-07-10

**Authors:** Yanjing Zheng, Yumei Li, Hongyan Long, Xiaojuan Zhao, Keke Jia, Juan Li, Leyong Wang, Ruiyong Wang, Xiancai Lu, Dongmei Zhang

**Affiliations:** ^1^State Key Laboratory of Pharmaceutical Biotechnology, School of Life Sciences, Nanjing University, Nanjing, China; ^2^State Key Laboratory for Mineral Deposits Research, School of Earth Sciences and Engineering, Nanjing University, Nanjing, China; ^3^Key Laboratory of Mesoscopic Chemistry of MOE and Collaborative Innovation Center of Chemistry for Life Sciences, School of Chemistry and Chemical Engineering, Nanjing, China

**Keywords:** *P. putida* MnB1, environmental stress, biofilm formation, anti-oxidative system, *mco*, *mnt*ABC, *sod*, *bifA*

## Abstract

*Pseudomonas putida* (*P. putida*) MnB1 is a widely used model strain in environment science and technology for determining microbial manganese oxidation. Numerous studies have demonstrated that the growth and metabolism of *P. putida* MnB1 are influenced by various environmental factors. In this study, we investigated the effects of hydrogen peroxide (H_2_O_2_) and manganese (Mn^2+^) on proliferation, Mn^2+^ acquisition, anti-oxidative system, and biofilm formation of *P. putida* MnB1. The related orthologs of 4 genes, *mco*, *mnt*ABC, *sod*, and *bifA*, were amplified from *P. putida* GB1 and their involvement were assayed, respectively. We found that *P. putida* MnB1 degraded H_2_O_2_, and quickly recovered for proliferation, but its intracellular oxidative stress state was maintained, with rapid biofilm formation after H_2_O_2_ depletion. The data from *mco*, *mnt*ABC, *sod* and *bifA* expression levels by qRT-PCR, elucidated a sensitivity toward *bifA*-mediated biofilm formation, in contrary to intracellular anti-oxidative system under H_2_O_2_ exposure. Meanwhile, Mn^2+^ ion supply inhibited biofilm formation of *P. putida* MnB1. The expression pattern of these genes showed that Mn^2+^ ion supply likely functioned to modulate biofilm formation rather than only acting as nutrient substrate for *P. putida* MnB1. Furthermore, blockade of BifA activity by GTP increased the formation and development of biofilms during H_2_O_2_ exposure, while converse response to Mn^2+^ ion supply was evident. These distinct cellular responses to H_2_O_2_ and Mn^2+^ provide insights on the common mechanism by which environmental microorganisms may be protected from exogenous factors. We postulate that BifA-mediated biofilm formation but not intracellular anti-oxidative system may be a primary protective strategy adopted by *P. putida* MnB1. These findings will highlight the understanding of microbial adaptation mechanisms to distinct environmental stresses.

## Introduction

Environmental stresses, such as nutrient depletion, extreme temperature and pressure, high salinity, strong ultraviolet light, and radiation, commonly induce microbes to produce cellular oxidative stress with overproduction of reactive oxygen species (ROS) (Teitzel and Parsek, 2003; Matallana-Surget et al., 2009; Chattopadhyay et al., 2011; Murata et al., 2011; Chen et al., 2013). Excessive ROS generation can injure proteins, DNAs and lipids, resulting in gene mutation and cell death (Cabiscol et al., 2000; Green and Paget, 2004; Lewis, 2008; Calabrese et al., 2011; Pan, 2011; Cap et al., 2012; Imlay, 2013; Chua et al., 2016). While low or moderate levels of ROS-induced physiological and biochemical changes are able to achieve an adaptation to the surrounding environment (Sies, 2017). Therefore, microorganisms have developed diverse mechanisms to respond to external ROS stimuli. For most microbial pathogens, the two primary mechanistic adaptations in response to ROS are excitation of anti-oxidative system (Apel and Hirt, 2004) and the formation of biofilms (Kubota et al., 2008). However, for non-pathogenic environmental microorganisms, the mechanistic basis for response to ROS (e.g., tolerance or scavenging) is less well understood.

Microbes in natural environments play important roles in global geochemical cycling, biodegradation of contaminants, and biofouling in the maintenance and evolution of local and global environments (Falkowski et al., 2008; Borch et al., 2010). ROS production in microbes during pathological condition is transient and moderate, while their exposure to consequent adverse factors in environments, results in oxidative stress (Cabiscol et al., 2000; Green and Paget, 2004; Matallana-Surget et al., 2009; Chattopadhyay et al., 2011; Murata et al., 2011; Chen et al., 2013; Imlay, 2013). Of note, microbes acquire energy by uptake of organic matter and redox reaction that take place on the transition metal ions in environment, sometimes producing secondary minerals (Rajkumar and Freitas, 2008; Soldatova et al., 2017). These physiological adaptation strategies allow microbes to survive in changing environments.

Manganese (Mn), as one of the most abundant transition metals in the earth’s crust, is encountered by microbes in the soil, water and atmosphere. In natural water, the concentration of Mn^2+^ ion ranges from 0.2 ∼ 3 mM (Humphris et al., 1996). Microbial Mn (II) oxidation is the major driving force in the biological formation of manganese oxide, controlling the Mn cycling in natural environments (Nealson et al., 1988; Handley and Lloyd, 2013). The direct oxidation and acquisition of Mn^2+^ are performed by manganese oxidase (MCO) (Brouwers et al., 1999, 2000; Francis and Tebo, 2001) and manganese transporter (Mnt) (Courville et al., 2006; Nevo and Nelson, 2006; Rees et al., 2009), respectively. In many microbes, Mn acts as a crucial trace nutrient for energy generation through Mn^2+^ oxidation (Cailliatte et al., 2010), which is influenced by diverse environmental factors, such as O_2_ levels, temperature, light, salinity and Mn^2+^ concentration (Hansel, 2017). Mn^2+^ in microbes also plays an important role in their adaptive response to intracellular and environmental condition changes (Kolenbrander et al., 1998; Loo et al., 2003; Papp-Wallace and Maguire, 2006; Juttukonda et al., 2016; Colomer-Winter et al., 2017; Qin et al., 2017), such as protections against oxidative damage (Latour, 2015) by functioning as a reducing reagent (Mahal et al., 2005), superoxide dismutase (SOD) mimic (Reboucas et al., 2008; Kelso et al., 2012) or SOD cofactor (Kim et al., 1999; Tseng et al., 2001). The uptake and utilization of Mn^2+^ by microbes favor adaptation to nutrient stresses (Cailliatte et al., 2010; Juttukonda et al., 2016) and defense against oxidative stresses (Kehl-Fie et al., 2013; Latour, 2015; Colomer-Winter et al., 2017). Modulation of Mnts balances Mn^2+^ availability, protects from the toxicity of excess Mn^2+^ (Qin et al., 2017), and affects microbial growth (Cailliatte et al., 2010), infection (Papp-Wallace and Maguire, 2006) and biofilm formation (Kolenbrander et al., 1998; Loo et al., 2003). Therefore, Mn oxidizing microbe is a good model for investigating the adaptive mechanisms when facing environmental stresses.

*Pseudomonas putida* (*P. putida*) MnB1, a prototype strain of the widely distributed *P. putida* species, has been widely used as a model strain for studying microbial Mn (II) oxidation in geochemical processes (Caspi et al., 1998; Villalobos et al., 2003; Toner et al., 2005; Parker et al., 2014). Numerous studies have been focused on the adsorption capacity of heavy metals and the formation of biogenic manganese minerals by *P. putida* MnB1 or *P. putida* GB1 (highly homologous to *P. putida* MnB1), which have successfully contributed to the environmental restoration of water, soil, and sediments (Villalobos et al., 2005; Sasaki et al., 2008; Meng et al., 2009; Forrez et al., 2011; Kim et al., 2012). Even though *P. putida* species are known to survive diverse exogenous stress factors, including heavy metal pollutants, superoxide (Lee et al., 2006; Park et al., 2006; Chavarria et al., 2013), antibiotics (Yeom et al., 2010), and organic compounds (Raiger-Iustman and Ruiz, 2008; Fernandez et al., 2012; Tavita et al., 2012; Lee et al., 2014), how *P. putida* MnB1 survives environmental stresses is not fully understood (Manara et al., 2012; Banh et al., 2013; Nikel et al., 2013; Ray et al., 2013).

It is known that *P. putida* biofilm formation correlates with adaptation and persistence in response to environmental stresses, in which cyclic diguanylate (c-di-GMP) signaling plays an essential role (Gjermansen et al., 2006; Matilla et al., 2011; Lee et al., 2016; Sun et al., 2017). c-di-GMP acts as a second messenger that modulates the planktonic to adhesive lifestyle switch, influencing virulence, infection and antibiotic resistance in pathogen (Romling et al., 2013). Generally, a rise in intracellular c-di-GMP promotes biofilm formation, while a decrease in c-di-GMP increases high exercise and dispersal (Wolfe and Visick, 2008; Borlee et al., 2010). Synthesis and hydrolysis of c-di-GMP are catalyzed by diguanylate cyclases (DGCs) and c-di-GMP-specific phosphodiesterases (PDEs), respectively. The role of c-di-GMP-specific PDE, BifA, in biofilm formation has been described in *P. putida* (Jimenez-Fernandez et al., 2015), *P. aeruginosa* (Kuchma et al., 2007), and *P. syringae* (Aragon et al., 2015). In these *Pseudomonas* species, *bifA* genes display a high degree of similarity and the proteins possess EAL domains that are essential for c-di-GMP-specific PDE activity. Carbon starvation is reported to induce biofilm collapse, which is directly related to *Bif*A PDE activity of *P. putida* (Gjermansen et al., 2005; Lopez-Sanchez et al., 2013). Δ*bifA* mutants increase biofilm formation in *Pseudomonas* species, and meanwhile exhibit reduction of starvation-induced biofilm dispersal in *P. putida* KT2442 (Jimenez-Fernandez et al., 2015), flagella-mediated motility in *P. aeruginosa* (Kuchma et al., 2007), and motility and virulence in *P. syringae* (Aragon et al., 2015). These observations indicate that c-di-GMP-specific PDEs are modulated by various environmental and/or intracellular signals to affect the microbe function (Gjermansen et al., 2005; Fang et al., 2014).

Several lines of evidence imply that the modulation of c-di-GMP-specific PDE closely interrelates with the cellular anti-oxidative system (Huang et al., 2013; Chua et al., 2016; Strempel et al., 2017; Wang et al., 2017), and responds to the presence of Mn^2+^ ion (Bobrov et al., 2005; Schmidt et al., 2005; Tamayo et al., 2005; Miner and Kurtz, 2016). Exposure to hydrogen peroxide (H_2_O_2_) for more than 120 generations increases rough and small colony variants (RSCV) in pathogenic *P. aeruginosa* (Chua et al., 2016). The appearance of RSCV, considered as a pre-biofilm form, increases microbial susceptibility to exogenous H_2_O_2_, which is restored by antioxidant L-glutathione treatment (Chua et al., 2016). Furthermore, H_2_O_2_ exposure leads to mutation in the *Wsp*F gene (encoded a c-di-GMP-specific PDE) that increases cellular c-di-GMP concentrations (Chua et al., 2016). Deletion of the *yjc*C gene (encoded a c-di-GMP-specific PDE) in *Klebsiella pneumoniae* increases sensitivity to H_2_O_2_ stress and decreases survival rate (Huang et al., 2013). Furthermore, ROS over-production causes the overwhelming formation of biofilms in *Klebsiella pneumoniae* CG43 (Huang et al., 2013). The phytopathogen *Xylella fastidiosa* with a mutation in the oxidative stress regulatory protein OxyR, exhibits more sensitive to H_2_O_2_ exposure but defective in biofilm maturation, suggesting that ROS may be an environmental cue to stimulate biofilm formation during host invasion (Wang et al., 2017). Therefore, c-di-GMP-specific PDE-mediated biofilm formation and intracellular anti-oxidative system may be closely associated with H_2_O_2_ stress. Moreover, c-di-GMP-specific PDE responds to Mn^2+^ ion (Bobrov et al., 2005; Schmidt et al., 2005; Tamayo et al., 2005; Miner and Kurtz, 2016) with significant effect on biofilm formation in pathogenic *Yersinia pestis* (Bobrov et al., 2005), *Vibrio cholera* (Tamayo et al., 2005), *Eschericia coli* (Schmidt et al., 2005), as well as in various environmental microbes, e.g., *Thermotoga maritima* (Miner and Kurtz, 2016).

In natural environments, microorganisms are commonly exposed to oxidative stresses (Cooper et al., 1988; Mopper and Zhou, 1990; Hakkinen et al., 2004; Kaur and Schoonen, 2017) in which a variety of substances (e.g., pyrite, manganese oxides, and pyrithione) spontaneously react with molecular oxygen to produce H_2_O_2_ (Borda et al., 2001; Schoonen et al., 2010; Kong et al., 2015), especially with the mediation of light (Mopper and Zhou, 1990; Zuo and Deng, 1999) and organic substances (Zuo and Hoigne, 1993). In this study, the effects of H_2_O_2_ and Mn^2+^ ion on growth, Mn^2+^ acquisition, anti-oxidative system and biofilm formation by *P. putida* MnB1 were assayed. A comparison of cellular response to H_2_O_2_ and Mn^2+^ ion in environmental microbes has the potential to provide insight into a universal mechanism to protect against exogenous stresses and adapt to changing environments.

## Materials and Methods

### Bacterial Strain

The *P. putida* MnB1 strain was provided by the China General Microbiological Culture Collection Center (CGMCC). *P. putida* MnB1 was cultivated in LEP medium (0.50 g yeast powder, 0.50 g acid hydrolyzed casein, 1.00 g glucose, 222 mg CaCl_2_, 0.39 g MgSO_4_, 1 mg FeCl_3_, 2.38 g HEPES (*N*-2-hydroxyethlpiperazine-*N*′-2-ethanesulfonic acid) (pH 7.5) per liter, containing 1 mL trace element solution (10 mg/L CaSO_4_⋅5H_2_O, 44 mg/L ZnSO_4_⋅7H_2_O, 20 mg/L CoCl_2_⋅6H_2_O, and 13 mg/L Na_2_MoO_4_⋅2H_2_O) (Boogerd and Devrind, 1987). The cultures were performed in 250 mL glass flasks at 30°C with shaking at 120 r/min.

### Cell Growth and Proliferation Monitoring

For measurement of cell growth, 25 mL cultures with a density of approximately 1 × 10^6^ cells/mL were incubated in LEP medium containing MnCl_2_ (0, 40, 200, 1000, and 5000 μM) or H_2_O_2_ (0, 40, 200, and 1000 μM) and monitored. The cultures were incubated with shaking at 30°C. The absorbance was measured at 600 nm (OD_600_) every 3 h for up to 48 h.

### H_2_O_2_ Scavenging Capacity

H_2_O_2_ concentrations remaining in cultures were assessed with a commercial H_2_O_2_ detection kit (Beyotime Institute of Biotechnology, Haimen, China). The detected maximum concentration of H_2_O_2_ in nature is 400 μM (Gunz and Hoffmann, 1990; Mopper and Zhou, 1990; Borda et al., 2001; Schoonen et al., 2010). Therefore, cells were incubated in LEP medium containing H_2_O_2_ (40, 200, and 1000 μM) for 24 h at 30°C with shaking. Cell cultures were harvested every 3 h and centrifuged (10,000 rpm, 30 s) and supernatants were collected. Following the protocol provided by the manufacturer, the absorbance at 560 nm was measured for detection of H_2_O_2_ concentration by the xylenol orange reaction.

Inactivated *P. putida* MnB1 was employed as a control to verify bio-degradation of the microbes on exogenous H_2_O_2_. *P. putida* MnB1 was inactivated in a water bath at 80°C for 30 min. H_2_O_2_ (1000 μM) was incubated in LEP medium with alive cells, inactivated cells, or in the absence of cells at 30°C with shaking. The culture supernatants were collected at 3 and 6 h, respectively. The xylenol orange reaction was conducted in polystyrene, flat-bottom, 96-well microplates in 3 replicates.

### Biofilm Observation and Qualification

Cell suspensions (1.5 mL) were inoculated in 6-well plates at a density of 1 × 10^6^ cells/mL. Coverslips and rhodochrosite slices (0.5 × 0.5 × 0.1 cm) were placed on the bottom of the plates and incubated at 30°C without shaking. After cell adhesion for 6 and 12 h, H_2_O_2_ (200 μM) was added to the cultures and incubated for another 2 h. Then coverslips and rhodochrosite slices were taken out and rinsed with phosphate buffer saline (PBS) for three times. Specimens were fixed with 2.5% glutaraldehyde and dehydrated in a graded ethanol series (30, 50, 70, 80, 90, and 100%) for 15 min each after rinsing 3 times. Finally, the samples were frozen at −80°C for 2 h and vacuum dried for 48 h. The samples were coated with platinum in a JEOL JFC-1600 auto fine coater device. Microbes colonized on glass coverslips or rhodochrosite slices were observed using a field emission scanning electron microscope (FESEM, Zeiss Supra55, Germany) at the State Key Laboratory for Mineral Deposits Research in Nanjing University.

Quantification of biofilm formation by *P. putida* MnB1 was conducted in polystyrene, flat-bottom, 96-well microplates (Thermo Scientific^TM^ Nunc^TM^ MicroWell^TM^ Cell-Culture Treated Microplates) with 5 replicates. A 100 μL (1 × 10^6^ cells/mL) cell suspension was incubated in LEP medium containing H_2_O_2_ (0, 40, and 200 μM) or MnCl_2_ (0, 40, 200, 1000, and 5000 μM) at 30°C without shaking for 6 and 12 h, respectively. After supernatants were removed, the biofilms were rinsed three times with PBS and stained with 100 μL of 0.1% (w/v) crystal violet (CV) for 15 min (Sule et al., 2009). CV was removed and the biofilms were rinsed three times before air drying. 100 μL of acetic acid (33%) was added to the biofilms, followed by a 30 min-incubation at 37°C to resolve CV completely. The solution was diluted (1:10) before measuring OD_590_ with a Safire Microplate Reader (Tecan Group Ltd., Mannedorf, Switzerland) to quantify the amount of CV absorbed by the biofilms.

The effect of H_2_O_2_ on biofilm formation after cell adhesion onto solid surfaces was investigated with a confocal laser scanning microscope (CLSM, Leica TCS SP8, Germany) at the State Key Laboratory of Pharmaceutical Biotechnology of Nanjing University. *P. putida* MnB1 cell cultures (1.5 mL) were inoculated into a 6-well plate at a density of 1 × 10^6^ cells/mL. A coverslip was placed on the bottom of the plate and incubated at 30°C for 6 and 12 h without shaking. After adhesion, H_2_O_2_ (0, 40, and 200 μM) was added to the culture for another 2 h. Then coverslips were taken out and rinsed three times with sterile deionized water. The colonized cells were stained with 1.0 mL of PBS containing 3.0 μL of STYO9 (3.34 mM) (Invitrogen, Carlsbad, CA, United States) for 20 min and washed three times with sterile deionized water. The prepared coverslips were fixed onto glass slides, and observed with the excitation and emission wavelength of 480 and 500 nm, respectively. The number of fluorescently labeled cells on the glass coverslips was quantified.

### Determination of Cellular Levels of ROS, SOD and Catalase Activity

For detection of catalase (CAT) activity in *P. putida* MnB1, 10 μL H_2_O_2_ (9.7 M) was added to a 10 μL cell suspension to observe bubble formation, in which H_2_O_2_ yielded H_2_O and O_2_, indicating the existence of CAT activity. Suspensions containing inactive cells (water bath at 80°C for 30 min), and cell culture after removing bacteria by 0.22 μm filtration were served as controls. Bubble formation was visualized by use of a Dissecting Microscope (Olympus SZ61 Stereo Microscope, Japan).

Total ROS was assessed with 10 μM 2′,7′-dichlorofluorescein diacetate (DCFH-DA, Beyotime Institute of Biotechnology, Haimen, China). 0.5 mL of cell suspension was placed in a 12-well plate at a density of 1 × 10^7^ cells/mL. Cell suspensions were incubated with LEP medium containing 200 μM H_2_O_2_ at 30°C without shaking for 3, 6, and 12 h. Following H_2_O_2_ treatment, the cells were harvested and incubated with DCFH-DA at 25°C for 30 min and then washed twice with PBS. The intensity of the fluorescent signal was analyzed with an excitation wavelength of 488 nm and monitoring the emission wavelength of 525 nm.

Following the H_2_O_2_ treatment similar to that of the ROS detection, cell lysates were harvested by liquid nitrogen freeze-thaw (three times) in lysis buffer (Beyotime Biotech, Nanjing, China). The supernatants were obtained by centrifugation (12,000 rpm, 3 min) to determine SOD and CAT activity (Beyotime Biotechnology, Haimen, China) by the WST-8 and the colorimetric method, according to the manufacturer’s instructions, respectively. The total ROS level, CAT, and SOD activity were calibrated and protein concentrations determined using a BCA protein assay kit (Beyotime Biotechnology, Haimen, China).

### Genomic DNA Extraction, Gene Amplification, and Alignment

The whole genomic DNA was isolated following the manufacturer’s instructions (BioTeke Corporation, Beijing, China). Cells in logarithmic phase (2 × 10^9^ cells in 2 mL) were centrifuged (10,000 rpm, 30 s) and the supernatants were discarded. After repeating centrifugation, the cells were re-suspended in 200 μL of Lysis Buffer A. 5 μL of lysozyme (10 mg/mL, dissolved in 10 mM Tris-HCl, pH 8.0) was added, mixed, and incubated at 37°C for 15 min. After the addition of 200 μL of Lysis Buffer B, the samples were immediately mixed and then treated with protease K (20 μL, 20 mg/mL) at 70°C for 10 min. After isopropanol precipitation and adsorption with Colum AC, the pellets were rinsed with Liquid Wash Buffer and then re-suspended in Liquid Elution Buffer. The extracted genomic DNA was preserved at −20°C for gene amplification.

Primers of the following gene orthologs – *mco*, *mntABC*, *sod*, and *bifA* were designed according to the GenBank whole genome of *P. putida* GB-1. The primers used for qRT-PCR were synthesized by SunShine Biotechnology (Nanjing, China) and listed in **Table 1**. The homology of gene and protein sequences (**Table 2**) were performed by using the alignment search algorithms BLASTN and BLASTX^1^ and compared to the encoded genes and proteins in *P. putida* KT2440 (NC_002947.4) and *P. putida* GB-1 (NC_010322.1).

**Table 1:** The primer sequences for qRT-PCR.

Gene		Primer sequences (5′→3′)
***mco***	Forward	ACGACGTCAACCTGGTGATA
	Reverse	TTGAGTTTGGGCTGGTACTG
***mntABC***	Forward	TCGAACAGGTCAGTTTCAGC
	Reverse	CAGCAGGGTCTTGATCAGC
***sod***	Forward	ACTGATGCGGTGAGTGATGT
	Reverse	CACTAGTGGTGCGTCGTTCT
***bifA***	Forward	CAGGTAACCCTGCTGGAAGT
	Reverse	GTGCACATTCTCGAAAGCAT

**Table 2:** The sequence similarity for the amplified PCR products by NCBI Blast.

Gene	Description	Identity	Accession no.
***mco***	***Gene:***		
	*Pseudomonas putida* strain PP112420, complete genome	99%	CP017073.1
	*Pseudomonas putida* GB-1, complete genome	99%	CP000926.1
	***Protein:***		
	Multispecies: multicopper oxidase (*Pseudomonas*)	100%	WP_012272302.1
***mntABC***	***Gene:***		
	*Pseudomonas putida* strain PP112420, complete genome	99%	CP017073.1
	*Pseudomonas putida* GB-1, complete genome	99%	CP000926.1
	***Protein:***		
	manganese ABC transporter ATP-binding protein (*Pseudomonas putida*)	100%	WP_012271765.1
	manganese ABC transporter ATP-binding protein (*Pseudomonas* sp. NBRC 111140)	99%	WP_060492768.1
***sod***	***Gene:***		
	*Pseudomonas putida* KT2440 chromosome, complete genome	95%	NC002947.4
	***Protein:***		
	Multispecies: superoxide dismutase (*Pseudomonas*)	100%	WP_012274023.1
	superoxide dismutase (*Pseudomonas* sp. URIL 14HWK12:14)	99%	WP_027609138.1
***bifA***	***Gene:***		
	*Pseudomonas putida* KT2440 chromosome, complete genome	89%	NC002947.4
	***Protein:***		
	Multispecies: cyclic diguanylate phosphodiesterase (*Pseudomonas*)	100%	WP_012272142.1
	diguanylate phosphodiesterase (*Pseudomonas* sp. NBRC 11114)	99%	WP_060493478.1

### RNA Extraction and Quantitative Reverse Transcription-Polymerase Chain Reaction (qRT-PCR) Analysis

Planktonic and colonized cells were collected from flask and 6-well plates, respectively. 1.0 mL of cell cultures (1 × 10^6^ cells/mL) were seeded into 100 mL LEP medium and incubated at 30°C with 120 r/min shaking. After 3, 6, 12, and 24 h of cultivation, 2.0 mL cell cultures were harvested by centrifugation at 10,000 rpm for 30 s. The precipitated cells were suspended and rinsed with PBS for three times. These planktonic cells were used for qRT-PCR assay. Cell cultures (1 × 10^6^ cells/mL in 1.5 mL) were seeded into 6-well plates and incubated at 30°C without shaking. After 3, 6, 12, and 24 h of cultivation, the upper culture medium and the floating cells were discarded, the adhesive cells were collected for qRT-PCR assay.

Modulations of gene expression in the biofilm cells treated with exogenous H_2_O_2_ and MnCl_2_ were assayed. Cells were seeded into 6-well plates (1 × 10^6^ cells/well) and incubated at 30°C without shaking to allow biofilm formation. After 6, 12, and 24 h of cultivation for adhesion, microbes were incubated with H_2_O_2_ (0, 200 μM) or MnCl_2_ (0, 200 μM) for another 2 h. The supernatants and the floating cells were removed to collect the adhesive cells for qRT-PCR assay.

Total RNA was isolated with Trizol reagent (Invitrogen, Carlsbad, CA, United States) according to the manufacturer’s instructions (*n* = 5/group). cDNA was synthesized in real time (RT) buffer with MultiScribe reverse transcriptase, and dNTPs (Promega, Madison, WI, United States). Relative mRNA expression was normalized to 16S rRNA. qRT-PCR analysis of mRNA was performed using SYBR Green I dye (Bio-Rad Laboratories, Hercules, CA, United States) according to the manufacturer’s protocol. The reaction mixture was incubated in a CFX96 Real-Time PCR Detection System (Bio-Rad) (30 s at 94°C followed by 45 cycles of 1 s at 94°C, 15 s at 55°C and 10 s at 72°C). Measurement of the fluorescence signal generated with SYBR Green I DNA dye was conducted at the annealing steps. Specificity of the amplification was confirmed by melting curve analysis. Data were collected and processed by CFX Manager Software (Bio-Rad), and expressed as a function of threshold cycle (Ct). The samples for qRT-PCR analysis were evaluated using a single predominant peak for quality control. The comparative Ct (2^−ΔΔC_t_^) method was used for analysis of relative mRNA expression, normalized to 16S rRNA.

### GTP Inhibition Evaluation

To investigate the role of BifA in *P. putida* MnB1, biofilm development was evaluated in the presence or absence of GTP, a potential inhibitor of c-di-GMP specific PDE (Ross et al., 1990; Barraud et al., 2009). Cells were seeded into 6-well plates (1 × 10^6^ cells/well) with and without 250 μM GTP pre-treatment. After 6 h or 12 h incubation, exogenous H_2_O_2_ (40, 200, and 1000 μM) or MnCl_2_ (40, 200, and 1000 μM) were added into the cell culture, respectively. After further incubation for 2 h, biofilm formation was validated by CV staining.

### Statistical Analysis

The data were presented as the mean ± SEM. Statistical analysis was performed using one-way analysis of variance (ANOVA), followed by the Dunnett’s Multiple Comparison Test. A value of *P* < 0.05 was considered statistically significant.

## Results

### Bacterial Growth and H_2_O_2_ Bio-Degradation

**FIGURE 1:**
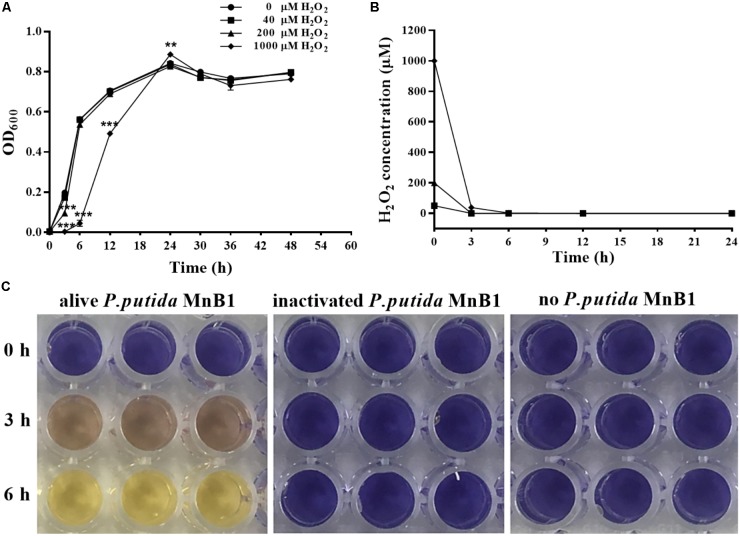
Exogenous H_2_O_2_ did not inhibit the growth of *P. putida* MnB1, but was bio-degraded quickly by *P. putida* MnB1. **(A)** Cells were incubated with 0, 40, 200, and 1000 μM H_2_O_2_, respectively. Growth of *P. putida* MnB1 was detected by measuring the absorbance at 600 nm (*n* = 3). **(B)** The concentration of H_2_O_2_ in culture with *P. putida* MnB1was assayed by the xylenol orange reaction (*n* = 3). **(C)** H_2_O_2_ concentration (initial concentration of 1000 μM) in LEP medium was assayed in the presence or absence of alive *P. putida* MnB1 or inactivated microbes, respectively. The supernatant of the cell culture was reacted with xylenol orange. Data were expressed as mean ± SEM. Significance, ^∗∗^*P* < 0.01 and ^∗∗∗^*P* < 0.001 vs. control group (0 μM H_2_O_2_).

As shown in **Figure 1A**, 40 μM H_2_O_2_ had little effect on proliferation of *P. putida* MnB1. With 200 μM H_2_O_2_, there was a slight retardation in proliferation during the early logarithmic phase. When H_2_O_2_ was increased to 1000 μM, proliferation during the first 24 h was remarkably suppressed, although biomass was quickly increased and then exceeded that of other groups at 24 h. H_2_O_2_ at the 3 tested dosages was degraded completely within 6 h by *P. putida* MnB1 (**Figure 1B**), whereas little H_2_O_2_ was decomposed within 6 h by dead cells or in medium alone (**Figure 1C**). Changes in proliferation indicated a defensive mechanism in *P. putida* MnB1 in response to exogenous H_2_O_2_. Since there was no significant change in proliferation of *P. putida* MnB1 at 40 and 200 μM of H_2_O_2,_ these working concentrations were employed for the following experiments.

### Biofilm Formation With Exogenous H_2_O_2_

Biofilm formation was investigated by CV staining. Based on preliminary data, biofilm formation could be detected before 3 h and the absorbance at OD_590_ was maintained steadily between 12 and 48 h. Therefore, most of our experiments were conducted before 12 h, which is defined as the earlier development stage before maturation (O’Toole and Kolter, 1998).

**FIGURE 2:**
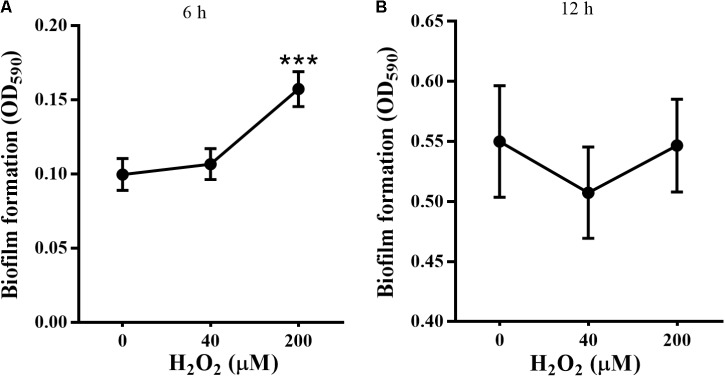
Effect of exogenous H_2_O_2_ on the biofilm formation by *P. putida* MnB1. Biofilm formation by *P. putida* MnB1 was quantified by CV staining with absorbance at 590 nm. Cells were incubated with H_2_O_2_ (0, 40, and 200 μM) for 6 h **(A)** and 12 h **(B)** (*n* = 6), respectively. Data were expressed as mean ± SEM. Significance, ^∗∗∗^*P* < 0.001 vs. control group (0 μM H_2_O_2_).

In this study, *P. putida* MnB1 was pre-cultivated in 96-well plates for 6 h or 12 h before exposure to H_2_O_2_. Biofilm biomass in the first 6 h was evidently promoted in the group with 200 μM H_2_O_2_ (**Figure 2A**). However, no distinct changes were observed at 12 h with 40 or 200 μM H_2_O_2_ exposure (**Figure 2B**). By integrating with the concentration changes of H_2_O_2_ within 6 h (**Figure 1B**), it was deduced that the promotion of H_2_O_2_ on the biofilm formation at earlier stage was pronounced.

**FIGURE 3:**
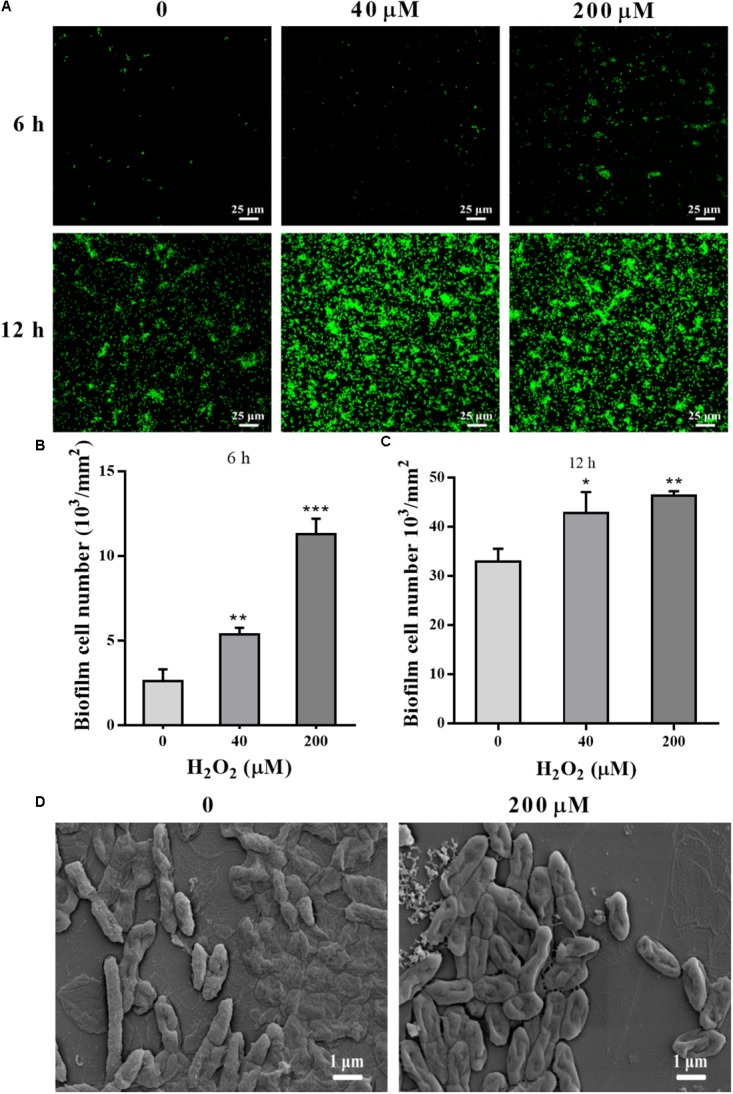
Confocal and FESEM observation of *P. putida* MnB1 biofilm formation with exogenous H_2_O_2_. After 6 and 12 h cultivation for adhesion, cells were incubated with H_2_O_2_ (0, 40, and 200 μM) for another 2 h, respectively. **(A)** Confocal observation of biofilm formation onto glass coverslips by STYO 9 staining (scale bar, 25 μm). Fluorescently labeled biofilm cells on the glass coverslips after incubation for 6 h **(B)** and 12 h **(C)** were quantified (field of vision, 276.79 × 276.79 μm) (*n* = 3). **(D)** FESEM images of *P. putida* MnB1 adhered to rhodochrosite with and without 200 μM H_2_O_2_ incubation for 12 h (scale bar, 1 μm). Data were expressed as mean ± SEM. Significance, ^∗^*P* < 0.05, ^∗∗^*P* < 0.01, and ^∗∗∗^*P* < 0.001 vs. control group (0 μM H_2_O_2_).

STYO 9 staining also showed that 40 and 200 μM H_2_O_2_ increased biofilm formation at 6 and 12 h on glass coverslips (**Figures 3A–C**). When the adhesion substrate was rhodochrosite, FESEM images showed nanowire development in H_2_O_2_-treated group besides of biofilm formation (**Figure 3D**).

### Intracellular ROS Levels and Anti-oxidative System With Exogenous H_2_O_2_ Treatment

**FIGURE 4:**
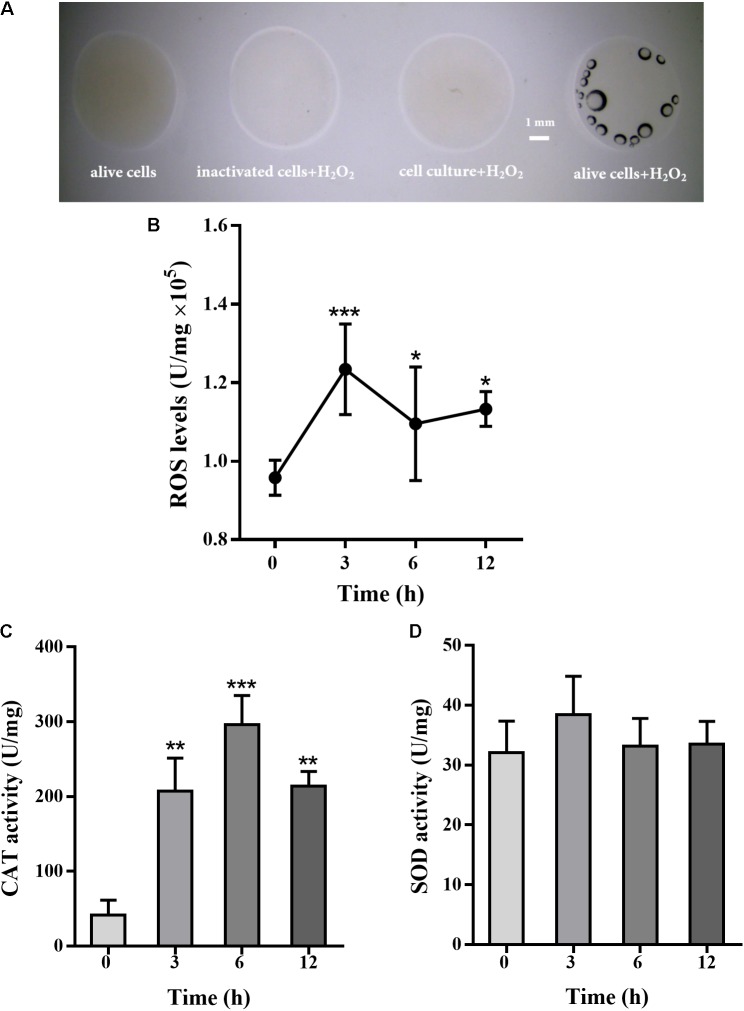
Effect of H_2_O_2_ on total cellular ROS levels, CAT and SOD activity in *P. putida* MnB1. H_2_O_2_ (9.7 M) was added to cell suspension, and bubble formation was observed by Dissecting Microscope (scale bar, 1 mm) **(A)**. The volume of four reactions were 20 μL, containing alive microbes (without H_2_O_2_), 10 μL cell culture after removing microbes and 10 μL H_2_O_2_, 10 μL inactivated bacteria with10 μL H_2_O_2_, and 10 μL alive microbes with 10 μL H_2_O_2_ (scale bar, 1 mm). Cellular total ROS levels were determined with 200 μM H_2_O_2_ incubation for 0, 3, 6, and 12 h (*n* = 4), respectively **(B)**. Cellular activity of CAT **(C)** and SOD **(D)** was analyzed, respectively (*n* = 4). Total ROS levels, CAT and SOD activity were calibrated to protein concentration in each sample. Data are expressed as mean ± SEM. Significance, ^∗^*P* < 0.05, ^∗∗^*P* < 0.01, and ^∗∗∗^*P* < 0.001 vs. control group (0 h incubation).

*Pseudomonas putida* MnB1 is a strict aerobe and possesses CAT. The activity of CAT was detected by oxygen bubble formation upon H_2_O_2_ addition. CAT activity was observed in alive *P. putida* MnB1, while no activity was detected in inactivated cells or culture medium after microbes removing (**Figure 4A**). Intracellular total ROS levels, when 200 μM H_2_O_2_ was added, were increased significantly at 3 h, and maintained until 12 h (**Figure 4B**). H_2_O_2_ exposure for 3, 6, and 12 h significantly increased CAT activity, but failed to change SOD activity (**Figures 4C,D**).

### *mnt*ABC, *sod*, and *bifA* Gene Modulation in Planktonic and Colonized Cells

Sequence similarity in nucleic acid and protein for the amplified products *mco*, *mntABC*, *sod*, and *bifA* was investigated by NCBI Blast. Most of the PCR products shared at least 98% homology with that of *P. putida* KT2440 or *P. putida* GB-1 (**Table 2**). The homology of each encoded protein predicted by NCBI Blast suggested the existence of conserved motifs.

**FIGURE 5:**
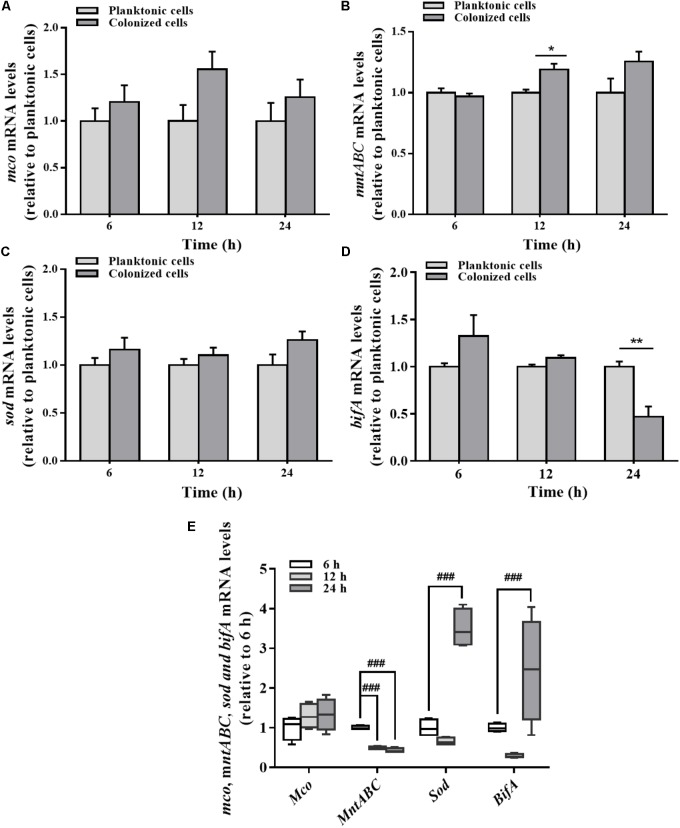
Expression of *mco, mntABC*, *sod*, and *bifA* gene between the planktonic and biofilm lifestyles as well as during biofilm development. *mco* **(A)***, mntABC* **(B)**, *sod* **(C)**, and *bifA* **(D)** mRNA levels in planktonic cells and colonized cells at 6, 12, and 24 h incubation were determined by qRT-PCR, respectively. **(E)** The cellular mRNA levels of *mco, mntABC*, *sod* and *bifA* during biofilm development in the colonized cells were determined by qRT-PCR. These expression levels were normalized to 16s rRNA (*n* = 4). Data were expressed as mean ± SEM. Significance, ^∗^*P* < 0.05, ^∗∗^*P* < 0.01, and ^∗∗∗^*P* < 0.001 vs. planktonic cell group. ^##^*P* < 0.01 and ^###^*P* < 0.001 vs. 6 h-biofilm formation group.

Differences in mRNA expression between planktonic and colonized cells were assayed by qRT-PCR. *mco* gene expression in the colonized cells was varied slightly at different stages relative to the planktonic cells (**Figure 5A**). No significant change in *mco* mRNA levels was detected during the stabilization and maturation of biofilms (**Figure 5E**). In contrast to the planktonic cells, *mntABC* mRNA levels were increased in colonized cells at 12 h (**Figure 5B**). However, *mntABC* gene expression was markedly decreased during biofilm maturation (**Figure 5E**).

There was no significant difference in the expression of *sod* gene between planktonic and colonized *P. putida* MnB1 cells (**Figure 5C**). But, *sod* mRNA levels were increased clearly when the biofilm was developed at 24 h (**Figure 5E**). Compared to planktonic cells, *bifA* gene expression in colonized cells was declined evidently, especially at 24 h (**Figure 5D**). *bifA* mRNA levels during the middle stage were lower than the earlier stage, but increased greatly at 24 h (**Figure 5E**).

### *bifA* and *sod* Gene Modulation by Exogenous H_2_O_2_

**FIGURE 6:**
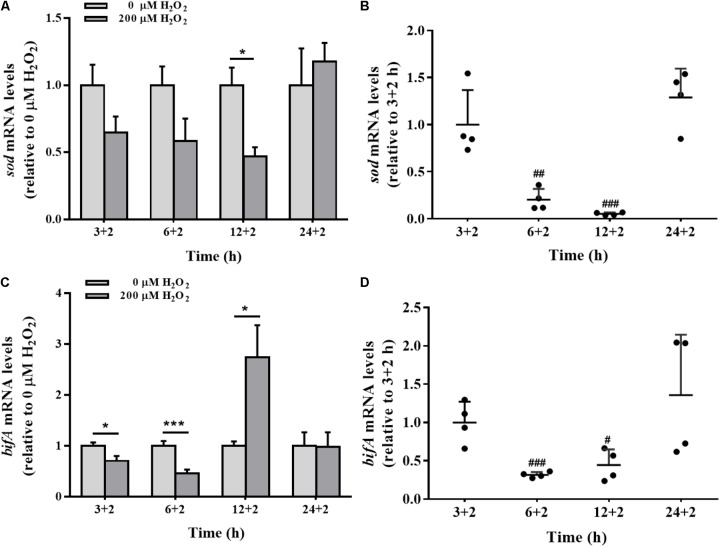
The *sod* and *bifA* mRNA levels in biofilm cells as well as during biofilm development with exogenous H_2_O_2_. *sod* **(A)** and *bifA* **(C)** mRNA levels were detected by qRT-PCR analysis in colonized cells incubated with 200 μM H_2_O_2_ for 2 h after 3 h, 6, 12, and 24 h cultivation for adhesion, respectively. Relative gene expression levels of *sod* and *bifA* were normalized to 16s rRNA (*n* = 4), respectively. The cellular mRNA levels of *sod* **(B)** and *bifA* **(D)** during different stages of biofilm formation. Data were expressed as mean ± SEM. Significance, ^∗^*P* < 0.05, ^∗∗^*P* < 0.01, and ^∗∗∗^*P* < 0.001 vs. control group (0 μM H_2_O_2_). ^#^*P* < 0.05, ^##^*P* < 0.01, and ^###^*P* < 0.001 vs. 3 h-biofilm formation group.

The effects of H_2_O_2_ on *sod* gene expression were time-dependent. Compared to the control group, H_2_O_2_ significantly down-regulated the expression of *sod* gene in biofilm cells at 12 h, with no apparent effect at 3, 6, or 24 h (**Figure 6A**). H_2_O_2_ significantly repressed *sod* mRNA levels at 6 and 12 h biofilm development, but such a repression was restored when the biofilm was developed to 24 h (**Figure 6B**).

The sensitivity of *bifA* gene expression modulation in biofilm cells under H_2_O_2_ exposure was evaluated at 3, 6, and 12 h. H_2_O_2_ significantly reduced the expression of *bifA* gene at 3 and 6 h, which were consistent with H_2_O_2_-promoted biofilm formation at the initial stage (**Figure 2A**). But, the decrease in the *bifA* mRNA levels was restored at 12 h of biofilm development (**Figures 6C,D**), being consistent with the CV staining results in which the effect of H_2_O_2_ on biofilm formation was nearly disappeared at that stage (**Figure 2B**). *bifA* gene expression was dysregulated by H_2_O_2_ throughout biofilm development.

### Bacterial Growth and Biofilm Formation With Exogenous Mn^2+^ Ion Supply

**FIGURE 7:**
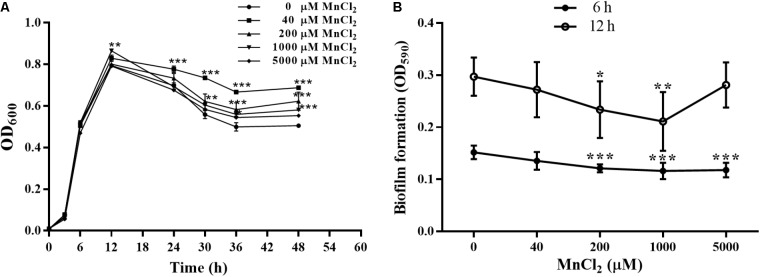
Effect of exogenous Mn^2+^ ion on the growth and biofilm formation by *P. putida* MnB1. **(A)** Microbial growth was detected by measuring the absorbance at 600 nm. *P. putida* MnB1 was incubated with 0, 40, 200, 1000, and 5000 μM Mn^2+^, respectively. Biofilm formation by *P. putida* MnB1 was quantified by CV staining with absorbance at 590 nm. Cells were incubated with Mn^2+^ (0, 40, 200, 1000, and 5000 μM) for 6 and 12 h (*n* = 6) **(B)**. Data were expressed as mean ± SEM. Significance, ^∗^*P* < 0.05, ^∗∗^*P* < 0.01, and ^∗∗∗^ *P* < 0.001 vs. control group (0 μM MnCl_2_).

Concentrations of 40, 200, 1000, and 5000 μM MnCl_2_ were selected as working dosages. As shown in **Figure 7A**, MnCl_2_ at all dosages had little effect on the proliferation of *P. putida* MnB1 within 12 h, but proliferation was promoted after the logarithmic growth period. Interestingly, 200 and 1000 μM MnCl_2_ were found to reduce biofilm formation significantly at 6 and 12 h (**Figure 7B**). Similar suppression effect was observed at 6 h with 5000 μM Mn^2+^ ion supply (**Figure 7B**).

### *mco, mnt*ABC, *sod*, and *bifA* Gene Modulation by Exogenous Supply of Mn^2+^ Ion

**FIGURE 8:**
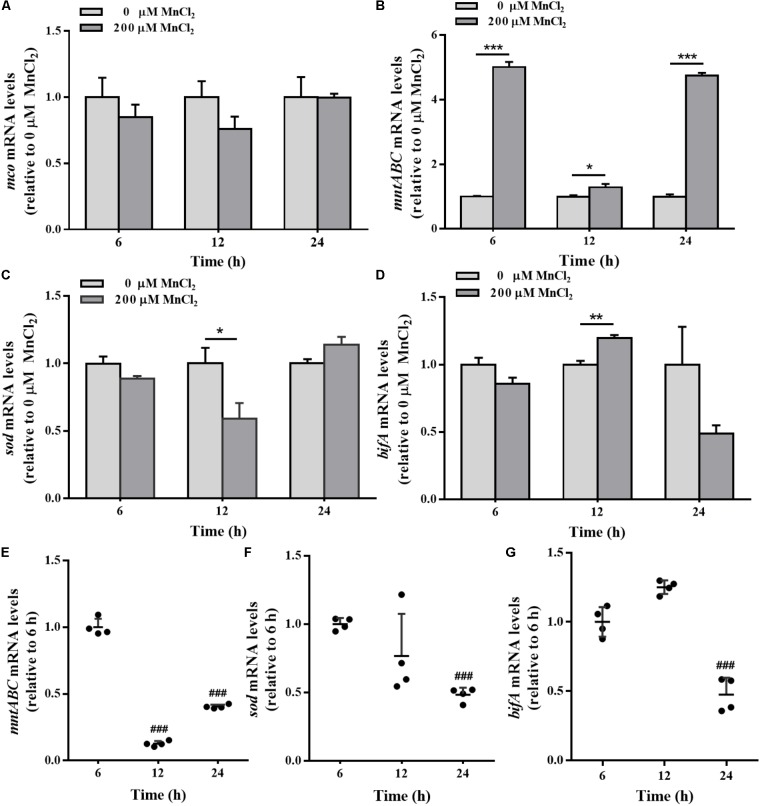
The *mco*, *mntABC*, *sod*, and *bifA* mRNA levels in biofilm cells as well as during biofilm development with exogenous Mn^2+^. *mco* **(A)***, mntABC* **(B)**, *sod* **(C)**, and *bifA* **(D)** mRNA levels in colonized cells incubated with 200 μM Mn^2+^ for 2 h after 6, 12, and 24 h cultivation for adhesion, were detected by qRT-PCR, respectively. The cellular mRNA levels of *mntABC* **(E)**, *sod* **(F)**, and *bifA* **(G)** at different stages of biofilm formation were detected by qRT-PCR, respectively. Relative gene expression levels of *mco, mntABC*, *sod* and *bifA* were normalized to 16s rRNA (*n* = 4), respectively. Data were expressed as mean ± SEM. Significance, ^∗^*P* < 0.05, ^∗∗^*P* < 0.01, and ^∗∗∗^*P* < 0.001 vs. control group (0 μM MnCl_2_). ^###^*P* < 0.001 vs. 3 h-biofilm formation group.

*mco* gene expression in the colonized cells was not sensitive to exogenous Mn^2+^ ion supply (**Figure 8A**). During biofilm development, the expression levels of *mntABC* in the colonized cells were remarkably up-regulated by 200 μM Mn^2+^ compared to planktonic cells (**Figure 8B**). Down-regulation of *sod* and up-regulation of *bifA* mRNA was also induced by 200 μM Mn^2+^ at 12 h (**Figures 8C,D**). Biofilm *mntABC* mRNA levels were decreased in the colonized cells at 24 h relative to 6 and 12 h (**Figure 8E**), indicating adaption to exogenous Mn^2+^ overload. Furthermore, *sod* mRNA levels were down-regulated, exactly similar to H_2_O_2_ exposure (**Figure 8F**). Additionally, *bifA* expression was increased at 12 h, but declined in biofilms at 24 h (**Figure 8G**) in the colonized cells exposed to 200 μM Mn^2+^.

### Regulatory Effect of GTP on Biofilm Formation

**FIGURE 9:**
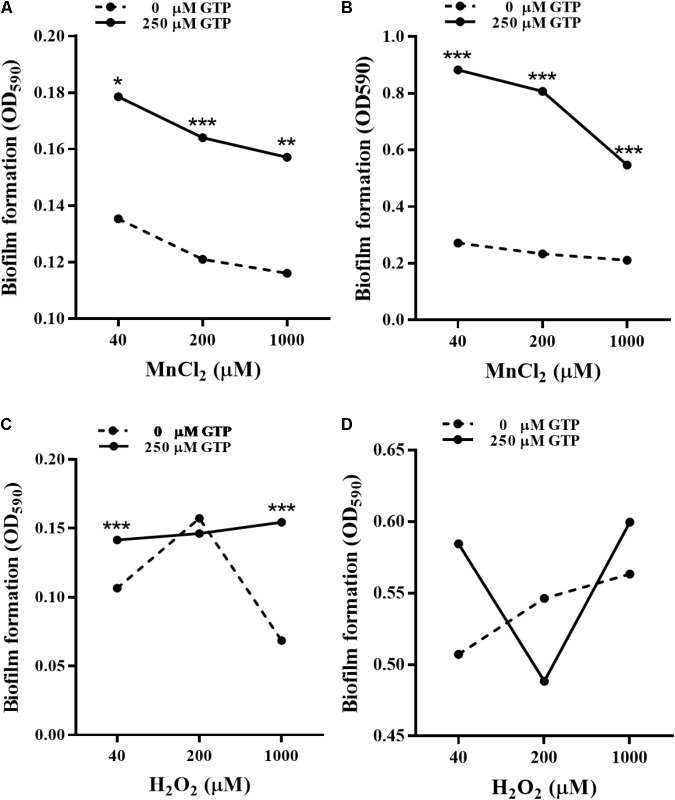
Effect of GTP on biofilm formation by *P. putida* MnB1. Microbes were incubated with Mn^2+^ at 40, 200, and 1000 μM for 6 h **(A)** and 12 h **(B)** in the presence or absence of 250 μM GTP (*n* = 6), respectively. Microbes were incubated with H_2_O_2_ at 40, 200, and 1000 μM for 6 h **(C)** and 12 h **(D)** in the presence or absence of 250 μM GTP (*n* = 6), respectively. Biofilm formation of *P. putida* MnB1 was quantified by CV staining with absorbance at 590 nm. Data were expressed as mean ± SEM. Significance, ^∗^*P* < 0.05, ^∗∗^*P* < 0.01, and ^∗∗∗^*P* < 0.001 vs. control group (0 μM GTP).

*bifA* mRNA levels in *P. putida* MnB1 were down-regulated by H_2_O_2_ at 3 and 6 h (**Figure 6C**), while the opposite modulation was observed with MnCl_2_ incubation at 12 h (**Figure 8D**). GTP is an effective inhibitor of BifA that partially blocks BifA-mediated degradation of c-di-GMP, promoting biofilm formation (Barraud et al., 2009). At different Mn^2+^ concentrations, pre-incubation with 200 μM GTP significantly promoted biofilm formation at 6 h (**Figure 9A**), this promotion was further strengthened to nearly 3–5 fold at 12 h (**Figure 9B**). GTP also increased biofilm formation at 6 h with 40 and 1000 μM H_2_O_2_ (**Figure 9C**). But, the amplification effect of GTP on biofilm formation did not show demonstrable at 12 h with H_2_O_2_ exposure at 3 dosages (**Figure 9D**).

## Discussion

In general, activation of anti-oxidative system (Kubota et al., 2008) and biofilm formation (Falkowski et al., 2008) are strategies utilized by pathogenic microorganisms to achieve stress tolerance. However, the mechanisms by which environmental microbes adapt to varying environments are not fully understood. In this study, the involvement of anti-oxidative system and c-di-GMP signaling in response to H_2_O_2_ and MnCl_2_ by *P. putida* MnB1 were investigated to elucidate the microbial adaptive mechanisms within natural environments.

### Modulation of Anti-oxidative System in Response to Exogenous H_2_O_2_

In this study, no cytotoxic effect of H_2_O_2_ (40–1000 μM) on *P. putida* MnB1 proliferation was observed. However, at 1000 μM H_2_O_2_, proliferation was retarded in the first 24 h (**Figure 1A**). Rapid recovery from high H_2_O_2_ levels suggests an effective defense mechanism against exogenous H_2_O_2_. *P. putida* MnB1 could quickly degrade H_2_O_2_ and produce O_2_ bubbles (**Figure 4A**), suggesting H_2_O_2_ decomposition (**Figures 1B,C**) by CAT activity (An et al., 2011; Chiang et al., 2011). However, cellular oxidative stress was maintained until 12 h even though the H_2_O_2_ had been scavenged (**Figures 1B,C**), with increased ROS levels (**Figure 4B**). Meanwhile, CAT activity increased significantly until 12 h (**Figure 4C**) induced by H_2_O_2_ exposure for decomposition. This decomposition improves cell viability by reducing oxidative damage to DNA and RNA in *Bifidobacterium* *longum* (Zuo et al., 2014) and in several UV-sensitive bacteria, during intracellular oxidative stress (Santos et al., 2012). During this procedure, *sod* over-expression synergistically accelerates the decomposition of exogenous H_2_O_2_ catalyzed by CAT. *Sod-*regulated anti-oxidative mechanisms are commonly adaptive processes for microbial protection (Green and Paget, 2004; Imlay, 2013). Intracellular ROS is produced not only physiologically, but also in response to environmental stimuli, including lack of nutrients, heavy metals contamination and ultraviolet light (Cabiscol et al., 2000; Green and Paget, 2004; Matallana-Surget et al., 2009; Chattopadhyay et al., 2011; Murata et al., 2011; Chen et al., 2013; Imlay, 2013). Herein, no significant SOD activation (**Figure 4D**) or *sod* up-regulation was observed during biofilm formation of *P. putida* MnB1 with H_2_O_2_ exposure (**Figure 6A**). In the presence of H_2_O_2_, *sod* up-regulation relieves oxidative stress and allows microbial adaptation to environmental change (Green and Paget, 2004; Imlay, 2013). Therefore, the function of SOD involved anti-oxidative system in the resistance to H_2_O_2_ in *P. putida* MnB1 is not observably crucial.

### Biofilm Formation in Response to Exogenous H_2_O_2_

In the present study, *bifA* gene expression was altered significantly when *P. putida* MnB1 cells were transformed from a planktonic to an adhesive lifestyle, as well as during the development of biofilms (**Figure 5D**). c-di-GMP specific PDEs modulate biofilm formation by decreasing cellular c-di-GMP levels as well as the sensitivity of microbes to environmental stresses (Osterberg et al., 2013; Fang et al., 2014; Aragon et al., 2015). It has been well disclosed that biofilm cells are more resistant to oxidative stress than planktonic cells in *phytopathogenic microbes* (Haque et al., 2017) and in animal pathogens (Hisert et al., 2005; Huang et al., 2013; Chua et al., 2016; Echeverz et al., 2017), which favors adaption and survival (Hisert et al., 2005; Dean et al., 2011; Wang et al., 2011; DePas et al., 2013; Chua et al., 2016). In this study, *bifA* gene expression (**Figures 6C,D**) and associated biofilm development (**Figures 2**, **3**) in *P. putida* MnB1 were clearly modulated by H_2_O_2_ exposure. Therefore, *bifA*-involved biofilm formation may be a defensive strategy utilized by *P. putida* MnB1 to survive H_2_O_2_ exposure-induced oxidative stress. Furthermore, the initiation of biofilm formation by *P. putida* MnB1 was more susceptible to H_2_O_2_ exposure than the mature biofilm (**Figures 6C,D**), being consistent with results obtained with *P. putida* KT2440 in response to ZnO nanoparticles (Ouyang et al., 2017). c-di-GMP-mediated adsorption is normally a key process for microbial leaching, in which biofilm formation is a defensive strategy against exogenous H_2_O_2_. 50 μM H_2_O_2_ promotes the adsorption of *Thiobacillus ferrooxidans* onto pyrite surfaces, enhancing the microbial oxidation of pyrite (Bellenberg et al., 2014). A significant increase in c-di-GMP content in colonized but not suspended cells has been reported in *T. ferrooxidans* (Ruiz et al., 2012).

It has been indicated that bacteria develop nanowires to facilitate efficient electron transport within the biofilm of microbial fuel cells (Reguera et al., 2005; Gorby et al., 2006) and clinical pathogens (Wanger et al., 2013). The nanowires formed between cells and the interface between cells and solid-phase, may contribute to biofilm development (Reguera et al., 2006, 2007), biofilm stabilization (Reguera et al., 2005; Gorby et al., 2006), and pathogenicity (Wanger et al., 2013). Acyl-homoserine lactone is a second messenger to regulate biofilm formation and triggers nanowires occurrence in *Aeromonas hydrophila* (Castro et al., 2014). Nanowires are proved to increase biofilm stabilization and decrease sensitivity to antibiotic treatment (Wanger et al., 2013). The nanowires observed in this study may exhibit the role of biofilm formation in response of *P. putida* MnB1 to H_2_O_2_ exposure (**Figure 3D**). This is an interesting observation that provides a foundation for further investigation of BifA-mediated biofilm development. Therefore, biofilm formation by *P. putida* MnB1 may defend against unfavorable environmental condition and it may be more sensitive than the intracellular anti-oxidative system.

### Mn^2+^ Ion Functions to Modulate Biofilm Formation

Effective acquisition of Mn^2+^ is normally involved in microbial resistance to oxidative stress and in bacterial pathogenesis (Papp-Wallace and Maguire, 2006; Coady et al., 2015). In this study, colonized cells exhibited an up-regulation of *mntABC* at mRNA level, suggesting the increased capacity for Mn^2+^ uptake (**Figure 5B**). Mn^2+^ overload increased *mntABC* gene expression as the biofilm was developed, demonstrating the adaptation potential (**Figure 8B**). Pathogens generally produce ROS for protection from the host immune response by increasing expression of Mn transporter proteins (Kehl-Fie et al., 2013; Park et al., 2017). Mutation or down-regulation of Mn transporters (MntABC, MntC, and MntH) significantly interferes with Mn^2+^ acquisition, increasing susceptibility to oxidative damage in *Streptococcus* (Wang et al., 2014; Chen et al., 2017), *Staphylococcus aureus* (Horsburgh et al., 2002; Coady et al., 2015), and *Neisseria gonorrhoeae* (Tseng et al., 2001; Kehl-Fie et al., 2013). SOD is an important oxygen free radical scavenger, and possibly use Mn^2+^ as a cofactor to influence disease progression (Miao and St Clair, 2009). MCO is the major Mn oxidation enzyme to supply energy for Mn-oxidizing microbes (Brouwers et al., 1999, 2000; Francis and Tebo, 2001; Dick et al., 2008). In the present study, *mco* levels were barely changed with exogenous Mn^2+^ (**Figure 8A**) and H_2_O_2_ (data not shown), indicating that Mn^2+^ ion supply did not remarkably affect Mn^2+^ oxidation. Similar to H_2_O_2_, exogenous Mn^2+^ ion decreased the expressions of *sod* significantly (**Figure 8C**), but negatively related to biofilm formation (**Figure 7B**). Mn^2+^ ion function to scavenge ROS as a cofactor for SOD, the effective acquisition of which is found to be closely associated with the repair of oxidative damage (DePas et al., 2013), thus ensuring the effective growth of bacteria after phagocytosis (Tseng et al., 2001; Horsburgh et al., 2002; Kehl-Fie et al., 2013; Wang et al., 2014; Coady et al., 2015; Chen et al., 2017).

As a type of trace nutrient, Mn^2+^ ion supply is an important factor affecting the biofilm formation (Shrout et al., 2006; Amaya-Gomez et al., 2015). In one respect, Mn^2+^ ion supply can promote the biofilm formation, which is restored by Mn^2+^ depletion in *Streptococcus mutans*, *P. aeruginosa*, and *Agrobacterium tumefaciens* (Shrout et al., 2006; Amaya-Gomez et al., 2015). In contrast, Mn^2+^ ion can inhibit biofilm formation by some microbes (e.g., *Yersinia pestis*), possibly through the activation of c-di-GMP specific PDE HmsP, being strictly dependent on Mn^2+^ (Bobrov et al., 2005). Suppression of *sod* demonstrates a ROS scavenger of Mn^2+^, which serves as a substitution for the protective role of biofilm formation. Consistently, *bifA* up-regulation was accompanied by biofilm suppression in *P. putida* MnB1 (**Figures 6**, **8**). Mutation of an oxidative stress regulatory protein, OxyR, makes cells more sensitive to H_2_O_2_, resulting in defective biofilm maturation in *Xylella fastidiosa*. Thus, ROS may be a potential environmental stimulus for biofilm formation during host invasion by the bacterial phytopathogen, *Xylella fastidiosa* (Wang et al., 2017), further suggesting a potential relationship between anti-oxidative system and biofilm formation. Therefore, biofilm formation may be a universal mechanism for adaptation to environmental changes, and Mn^2+^ ion may decrease biofilm formation through regulation of *bifA* in *P. putida* MnB1.

### BifA-Involved Biofilm Formation: A Sensitive Strategy for Protection

**FIGURE 10:**
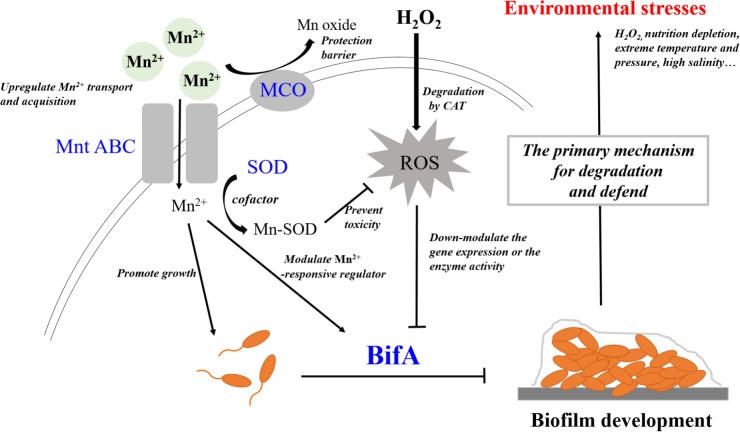
BifA regulated-biofilm formation is a primary mechanism to defend exogenous H_2_O_2_ exposure and closely correlates to cellular oxidative stress. Mn^2+^ ion and H_2_O_2_ represent 2 types of environmental factors, a trace nutrient substrate and an oxidative stress, respectively. Four genes that encode MCO, Mn^2+^ transporter, SOD and BifA were assayed for their expression during bacterial growth following exposure to the two environmental factors Mn^2+^ ion and H_2_O_2_ on the growth, Mn^2+^ acquisition, anti-oxidative system and biofilm formation in *P. putida* MnB1. Exogenous Mn^2+^ promoted Mn^2+^ uptake and acquisition. An increase in intracellular Mn^2+^ may act as nutrient substrate promoting microbial growth. Alternatively, Mn^2+^ may function as a cofactor for important enzymes, such as SOD, which scavenges ROS. Mn oxide, catalyzed by MCO is a barrier to protect from invasion, heavy metal and oxidative damage. Mn^2+^ may target Mn^2+^-responsive regulators to inhibit biofilm formation by BifA up-regulation. Decrements in intracellular anti-oxidative system and the inhibition of biofilm formation suggests that Mn^2+^ favors the microbial growth but suppresses biofilm development. Exogenous H_2_O_2_ triggers intracellular ROS over-production, but does not significantly affect the proliferation of *P. putida* MnB1. Bio-degradation of exogenous H_2_O_2_ and quick recovery of proliferative capacity at high H_2_O_2_ levels are due to CAT activity. In contrast to the activation of anti-oxidative system, promotion of the biofilms by down-regulation of *BifA* gene or the blockade of BifA activity suggests that biofilm formation may be a primary mechanism by which *P. putida* MnB1 defends unfavorable environmental factors. Therefore, BifA-mediated biofilm formation may be more sensitive than anti-oxidative system in response to Mn^2+^ ion and H_2_O_2_ in *P. putida* MnB1. The sensitivity of BifA-mediated biofilm formation may highlight its role in the adaption of microbes to environment stress. MCO, Manganese oxidase; MntABC, ABC-Type manganese transporter; SOD, Superoxide dismutase; CAT, Catalase; PDE, Phosphodiesterase; ROS, Reactive oxygen species.

Mn^2+^ and H_2_O_2_ can be considered a trace nutrient supply and an environmental stress for *P. putida* MnB1, respectively. A close relationship was observed between regulation by Mn^2+^ and H_2_O_2_ and the formation and development of biofilms (**Figures 2**, **3**, **7**). Moreover, GTP reversed the suppressive effect of Mn^2+^ on biofilm formation and greatly increased biofilm development for *P. putida* MnB1 (**Figures 9A,B**). GTP also accelerated biofilm formation at the initiation stage following the addition of H_2_O_2_ (**Figures 9C,D**). A *bifA* ortholog was amplified from the *P. putida* MnB1 genome, sharing 89% homology with the c-di-GMP PDE of *P. putida* GB1. Analysis with the alignment search algorithm BLASTX showed that the *P. putida* MnB1 BifA ortholog contain an EAL motif, which is crucial for c-di-GMP specific PDE activity. DGC synthesizes c-di-GMP in a GTP-dependent manner (Chen and Schaap, 2012) and c-di-GMP specific PDE is highly responsive to intracellular GTP availability (Christen et al., 2005; Barraud et al., 2009; An et al., 2010; Purcell et al., 2017). In response to diverse stresses on *P. aeruginosa*, c-di-GMP specific PDE mediates biofilm formation as well as the production and secretion of virulence factors that play a vital role in escape from host defense. In *P. aeruginosa*, biofilm dispersal and intracellular c-di-GMP specific PDE activity are significantly abrogated in the presence of GTP (Barraud et al., 2009). The gene *rbdA* encodes a bifunctional protein containing highly conserved DGC (GGDEF) and c-di-GMP specific PDE (EAL) motifs. GTP increases the c-di-GMP specific PDE activity of RbdA by allosterically modulating the GGDEF domain, promoting biofilm dispersal and production of virulence factors (rhamnolipids and exopolysaccharides) (An et al., 2010). Consistent with the results from *P. aeruginosa* (Barraud et al., 2009), we also manifested that GTP improved biofilm formation of *P. putida* MnB1, suggesting a key role for BifA in the formation of *P. putida* MnB1 biofilms as a sensitive defense to environmental stresses (**Figure 10**).

Deletion of the *yjc*CT gene of *Klebsiella pneumoniae* CG43 (a c-di-GMP specific PDE), promotes sensitivity to H_2_O_2_ treatment, with the reduction of survival rate. At the same time, ROS overproduction generally accompanies biofilm development (Huang et al., 2013). For the pathogenic bacteria *P. aeruginosa*, exogenous H_2_O_2_ promotes biofilm formation (Chua et al., 2016). Furthermore, mutation in *Wsp*F is induced by H_2_O_2_, with high intracellular c-di-GMP concentration and biofilm development (Chua et al., 2016). *cdg*R encodes a c-di-GMP specific PDE in *Salmonella enteric* var. Typhimurium, interference of which decreases resistance to H_2_O_2_ (Hisert et al., 2005). Therefore, *bifA* can respond to environmental factors by regulating biofilm formation, which is more sensitive than the intracellular anti-oxidative system in *P. putida* MnB1.

## Conclusion

In this study, the effects of H_2_O_2_ and Mn^2+^ ion on *P. putida* MnB1 growth, Mn^2+^ ion acquisition, anti-oxidative system, and biofilm formation were investigated. Exogenous Mn^2+^ ion supply promoted the growth and Mn^2+^ uptake capacity of *P. putida* MnB1, but suppressed biofilm formation. Exogenous H_2_O_2_ was bio-degraded quickly in the presence of *P. putida* MnB1, with maintained cellular oxidative stress after H_2_O_2_ depletion. No significant SOD activation or *sod* gene up-regulation was detected in *P. putida* MnB1with H_2_O_2_ exposure. In contrast, *bifA* gene expression and subsequent biofilm formation were significantly modulated by Mn^2+^ ion and H_2_O_2_. The correlation between *bifA-*mediated biofilm formation and effect of Mn^2+^ ion and H_2_O_2_ was further manifested by blocking BifA activity in the presence of GTP. Sensitivity differences between intracellular anti-oxidative system and biofilm formation suggests that BifA-mediated biofilm formation may be a primary defense mechanism by *P. putida* MnB1 in response to environmental factors. These findings highlight the role of biofilm development in adaption of microbes to environment stresses.

## Author Contributions

DZ conceived and designed the study. YZ conducted the experiments and prepared the manuscript. YL, HL, XZ, and KJ conducted the experiments. JL, LW, and RW analyzed and interpreted the data. DZ and XL wrote and revised the manuscript.

## Conflict of Interest Statement

The authors declare that the research was conducted in the absence of any commercial or financial relationships that could be construed as a potential conflict of interest.

## Footnotes

1 https://blast.ncbi.nlm.nih.gov/Blast.cgi
